# [Retracted] MicroRNA-155 promotes tumor growth of human hepatocellular carcinoma by targeting ARID2

**DOI:** 10.3892/ijo.2026.5886

**Published:** 2026-04-22

**Authors:** Li Zhang, Wei Wang, Xiaobin Li, Sai He, Jianfeng Yao, Xiaolong Wang, Di Zhang, Xuejun Sun

Int J Oncol 48: 2425-2434, 2016; DOI: 10.3892/ijo.2016.3465

Following the publication of the above paper, a concerned reader drew to the Editor's attention that, in addition to concerns about the description of one of the antibodies employed in this study, the immunohistochemical data shown in Fig. 4E on p. 2429 and western blot data shown in Fig. 6E on p. 2431 had apparently appeared in figures in other papers that had already been submitted to the journals *Oncotarget* and I*nternational Journal of Oncology* respectively, both of which were written by different authors at different research institutes. Upon performing an independent analysis of the data in the Editorial Office, we were able to validate these concerns; moreover, some of the same western blot data had also apparently been included in Figs. 4 and 5.

Given that the abovementioned data had already been submitted for publication to other journals, the Editor of *International Journal of Oncology* has decided that this article should be retracted from the Journal. The authors were asked for an explanation to account for these concerns, but the Editorial Office did not receive a reply. The Editor apologizes to the readership for any inconvenience caused.

